# Authors’ reply

**Published:** 2011

**Authors:** Nita Umesh Shanbhag, Sumita Karandikar, Pooja Anil Deshmukkh

**Affiliations:** Department of Ophthalmology, DY Patil Medical College & Research Center, Nerul, Navi Mumbai, Maharashtra, India

Dear Editor,

We thank Rath *et al*.[1] for the interest shown in our article[2] and their comments.

They are correct in their supposition that the patient reported by us and Patil *et al*. is the same.

To quote from our article, “We are reporting this case because orbital involvement of actinomycetoma is very unusual. In our review of the literature, we did not come across any such case.” We do not claim to be the first to report a case of orbital actinomycetoma. Our literature search did not bring up the articles by Pagliani *et al*.[3] and Sullivan *et al*.[4] and we thank Rath for correcting our lapse. This patient was first seen and primarily treated at the ophthalmology department, and inputs were obtained from the dermatology department for the skin lesions and the pharmacological treatment.

At the time of submission of our article (September 2008), we were not aware that this article was to be submitted by the dermatology department (Patil *et al*.[5]). Our article as it turns out took much longer in the editorial process, and was eventually published only in January 2010. As far as we could ascertain, they have highlighted the dermatology points and we have focused on the ophthalmic aspects.
